# Do not go gently into that good night: The dying brain and its paradoxically heightened electrical activity

**DOI:** 10.1073/pnas.2305985120

**Published:** 2023-05-22

**Authors:** Christof Koch

**Affiliations:** ^a^MindScope Program, Allen Institute, Seattle, WA 98109; ^b^Tiny Blue Dot Foundation, Santa Monica, CA 90401

No one gets out of this life alive. Although about three million Americans die each year, science and medicine still know remarkably little about the underlying processes, in particular what happens to the brain, the organ of consciousness, in its final minutes. This territory is explored in PNAS by Xu et al. (1) from the University of Michigan School of Medicine.

There are, of course, many ways to die. The Uniform Determination of Death Act, the way death is established in the United States, distinguishes cardiopulmonary death—the lungs stop breathing and the heart stops beating—from irreversible whole-brain death. Most who die follow the cardiopulmonary route (2).

A common example is cardiac arrest that annually kills more than 400,000 in the United States. With the pulse of life gone, the brain quickly shuts down due to loss of oxygen. Within seconds, the victim faints, that is, loses consciousness. The brain’s electrical activity, as assayed by scalp electroencephalography (EEG) electrodes, rapidly diminishes until all activity ceases and the EEG becomes iso-electric or flat. Indeed, “EEG suppression” is a universal sign of deep coma and impending death. At this point, the mind is extinguished—no more experiencing, thinking, fearing, hoping, or remembering.

However, while this view of dying makes intuitive sense, the brain can marshal resources that belie such a simple narrative. Indeed, there have been persistent reports in the clinical literature of resurging EEG activity that occurs minutes after loss of blood pulse. These end-of-life EEG surges were initially believed to be artifacts but are now recognized as reflecting high-frequency brain activity. They are common (46% in ref. 3) in critically ill patients who die but, importantly, are not found in brain-dead patients. Experiments in anesthetized rats, in which death was induced by injection of KCl into the heart, likewise revealed a paradoxical and transient increase in synchronous high-frequency electrical activity within the first half minute following cardiac arrest before flat-lining (4). Activity in the spectral range of 25 to 150 Hz, the so-called gamma oscillations, of the EEG and the local field potential in the cortex is thought to be one of the essential hallmarks of consciousness in mammals, including in people (5).

Xu et al. analyzed the EEG and EKG records of four neurological intensive care unit (NICU) patients, all of whom were behaviorally unresponsive, with eyes closed and no signs of overt consciousness. Given their poor prognosis, and in consultation with family members, life support was discontinued. All comatose patients died, with the final heartbeat detected within 10 to 25 min of ventilator removal.

**Fig. 1.**
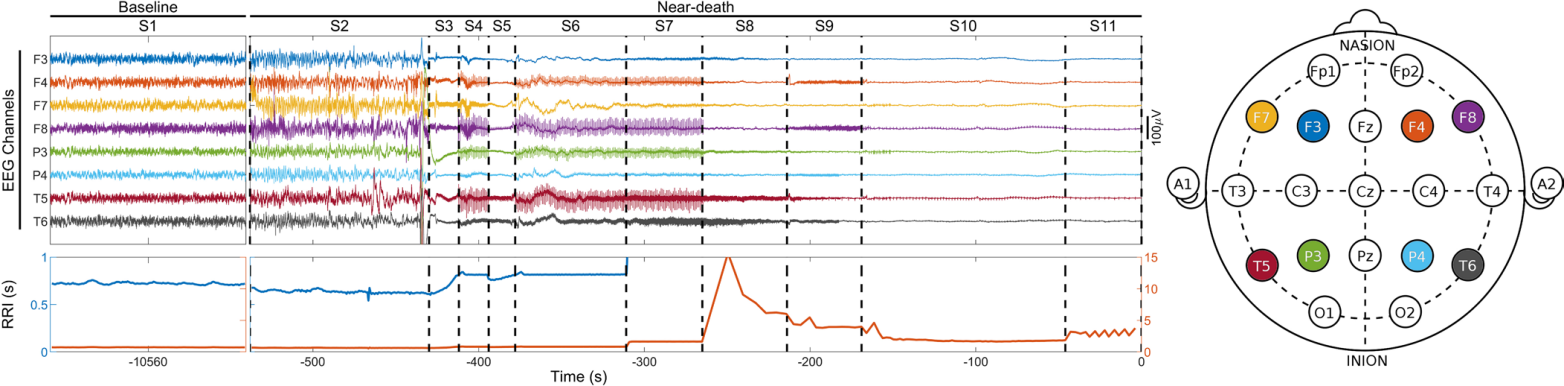
Select EEG channels from a 24-y-old comatose woman with cardiac arrest–induced anoxic injury on life support (S1 or baseline). The ventilator was removed at the start of S2, at which point high-frequency and high-amplitude activity develops. S2 ends with EEG suppression. The patient’s final heartbeat occurs at the end of S11, on the right. The location of the eight EEG electrodes, out of 19, is illustrated in the schematic on the right. The duration of heartbeat intervals (RRI) is shown on the bottom on a short (blue, on the left; 0 to 1 s) and long (brown, on the right; 0 to 15 s) timescales. Modified from figure S1A in Xu et al. (1).

The clinicians–scientist team used the unprecedented opportunity of having a high-quality EEG montage in place to learn more about the twilight zone between life and death (see ref. 6 for one other EEG analysis of a dying patient). They segmented the time between ventilator turnoff and the last heartbeat into stages (S2, S3, and so on) based on cardiac activity, relative to activity at baseline prior to S1. In one patient (see figure), periods of slowing heart rate and reduced EEG amplitude (S3 and S5) alternate with periods of pacemaker-associated heart rate stabilization and an enhanced EEG with higher frequency activity (S4, S6, and S7), indicative of two-way interactions between the brain and the heart (Fig. 1).

What is quite apparent is that loss of oxygen triggers hyperexcitability, with broad increases in beta and gamma band activity in frontal and central cortical regions in two out of four patients. The same two showed subsequent episodes of recurrent large beta and gamma activity in the temporal lobes, involving the somatosensory cortex (SSC). The amplitude of the high-frequency gamma activity was coupled to the phase of the activity in the slower beta band between the dorsolateral prefrontal cortex and the SSC, compatible with communication between these regions (7).

Even more remarkable, a measure of directed functional connectivity in the gamma range implicated a region in the temporo–parietal–occipital cortex as being highly interconnected. This region is part of the posterior hot zone, thought to be the neural substrate of consciousness (8). For more details regarding the spatial–temporal organization of this activity pattern and its evolution across the dying process, consult the paper.

Xu et al. provide strong evidence that the dying brain is not necessarily the quiet place, electrically speaking, that it was thought to be.

The EEG of the other two patients showed no rapid activity but only the large and slow waves characteristic of deep unconsciousness that morphed within a few minutes into a flat line.

The authors argue that their data are compatible with two patients awakening from their terminal coma by the alarming drop of oxygen (hypoxia) and consciously experiencing something before death. They speculate that these experiences might be similar to the feelings of peace and transcendence reported during near-death experiences by survivors (9). Perhaps.

However, there are several possible confounds. While the authors judiciously address these concerns, they are up against hard methodological constraints working with severely impaired patients in an NICU environment.

The first one is increased muscle tone during the dying process. Using an independent component analysis, the authors identify high-frequency contamination in the EEG by muscular activity. Yet these muscle artifacts appear to be insufficient to fully explain their data.

A more serious concern is that the massive increase in gamma power (up to a factor of 392) is compatible with epileptic seizures. Triggered by anoxia and ischemia, ionic gradients of individual neurons are running down and massive neurotransmitter release is happening, all under extreme pathological conditions that perturb the fine balance between excitation and inhibition. This is a perfect breeding ground for hypersynchronized neuronal assemblies, localized to specific cortical neighborhood.

My confidence that these signals are footprints of consciousness would be increased if such large gamma surges in the posterior hot zone could be observed in neurotypical subjects, for example during unusual experiential states that share some characteristic with near-death experiences, such as high doses of psychedelics that can lead to ego dissolution and mystical experiences (10). A more direct way to detect covert consciousness in behaviorally unresponsive neurological patients probes the cortex with magnetic pulses and listens to the echoes using high-density EEG (11).

No matter the outcome, Xu et al. (1) provide strong evidence that the dying brain is not necessarily the quiet place, electrically speaking, that it was thought to be. Investigating the neural correlates of dying is not only a scientific problem but touches on existential concerns, with clinical and ethical implications for literally everyone. Sooner or later, all of us might discover for ourselves whether the dying brain is capable, however briefly, of experiencing something before it crosses the fundamental divide between being and nonbeing.
